# HER2-Low Breast Cancer: Biological Framework and Determinants of HER2 Instability

**DOI:** 10.3390/medicina62020304

**Published:** 2026-02-02

**Authors:** Alina-Mihaela Gurau, Daniela Mihalache, Catalin-Bogdan Satala, Ana Maria Rață, Laura-Florentina Rebegea

**Affiliations:** 1 The School for Doctoral Studies in Biomedical Sciences, “Dunărea de Jos” University of Galați, 800008 Galați, Romania; alinamihaelagurau@gmail.com (A.-M.G.);; 2 Faculty of Medicine and Pharmacy, Medical and Pharmaceutical Research Center, “Dunărea de Jos” University of Galați, 800008 Galați, Romania; 3 Department of Pathology, Clinical County Emergency Hospital Braila, 810325 Braila, Romania; 4 Radiotherapy Department, Emergency Hospital “Sf. Apostol Andrei” Galați, 800578 Galați, Romania; 5 Medical Clinical Department, Faculty of Medicine and Pharmacy, “Dunarea de Jos” University, 800008 Galați, Romania; 6 Research Center in the Field of Medical and Pharmaceutical Sciences (ReFORM-UDJ), 800010 Galați, Romania

**Keywords:** HER2-low, breast cancer, intratumoral heterogeneity, antibody-drug conjugate, biomarker reassessment

## Abstract

Human epidermal growth factor receptor 2 (HER2)-low breast cancer is a clinically relevant subgroup defined by low but detectable HER2 protein expression, immunohistochemistry (IHC) score of 1+ or 2+ with negative in situ hybridization findings, positioned at the interface between traditional HER2-positive and HER2-negative disease. The recent introduction of antibody–drug conjugates (ADCs) has increased the clinical significance of borderline HER2 expression and exposed important diagnostic challenges, particularly in cases with very low levels of membrane staining, including the emerging HER2-ultralow category. *Background and Objectives*: This review summarizes the pathological and biological framework of HER2-low and HER2-ultralow breast cancer and critically appraises the magnitude, direction, and determinants of HER2 variability under systemic therapy. Particular focus is placed on treatment-associated shifts after chemotherapy, intratumoral heterogeneity, and pre-analytical and analytical factors that can influence HER2 assessment, with direct implications for therapeutic stratification and biomarker reassessment. *Materials and Methods*: A narrative literature review was conducted using PubMed, Scopus, and Web of Science, focusing on studies published within the last five years. Eligible publications included clinical trials, retrospective cohorts, and translational or molecular studies that reported paired HER2 assessment in breast cancer and were interpreted according to American Society of Clinical Oncology/College of American Pathologists-aligned criteria. *Results*: Across major cohorts, HER2-low appeared to be the most dynamic category, with variability frequently observed following systemic therapy. Beyond treatment-related effects, shifts in HER2 status may be attributable to intratumoral heterogeneity and technical variability, with the greatest impact observed at the IHC 0–1+ interface. *Conclusions*: Given the clinical relevance of low-level HER2 expression, standardized testing and transparent reporting are essential, and HER2 reassessment may be justified in selected clinical scenarios to optimize access to HER2-directed therapies.

## 1. Introduction

Breast cancer remains a major global health issue and a heterogeneous disease in terms of its biological, histopathological, and molecular features [1]. In clinical practice, stratification according to hormone receptor (HR) and HER2 status is fundamental for prognosis and treatment selection. Over the past decade, advances in accurately determining HER2 status have enabled clinicians to identify patients eligible for targeted anti-HER2 therapies, thereby personalizing treatment and significantly improving clinical outcomes [2,3,4,5].

A substantial proportion of tumors historically classified as HER2-negative show low-to-intermediate HER2 protein expression, exhibiting an immunohistochemistry (IHC) score of 1+ or 2+ and negative in situ hybridization (ISH) findings. These tumors, referred to as HER2-low, are considered a therapeutically relevant subset within the traditional HER2-negative category, although they do not constitute a single, uniform biological entity [2,3,4,5,6,7,8,9]. Recently, particularly since the emergence of dedicated analyses in 2020, HER2-low has become an established term in the literature, as the clinicopathological features and prognostic impact of this subtype have been investigated more systematically [7,8,9,10]. The development of novel antibody–drug conjugates (ADCs), especially trastuzumab deruxtecan (T-DXd), has been pivotal. In 2022, the phase III DESTINY-Breast04 trial showed that T-DXd significantly improved progression-free and overall survival rates compared with the physician’s choice treatment in patients with unresectable or metastatic HER2-low breast cancer. These findings established HER2-low disease as a clearly targetable subgroup and changed the standard of care in this setting [10,11,12,13,14,15]. More recently, the emerging term HER2-ultralow has been used to describe tumors with borderline or extremely low HER2 expression below the HER2-low threshold, further highlighting the clinical significance of the low-expression spectrum [16,17,18,19,20,21].

Beyond the simple HER2-positive versus HER2-negative dichotomy, HER2 expression is increasingly recognized as a dynamic rather than fixed feature of breast cancer, and similar intratumoral heterogeneity and temporal variability have been described in other tumor types, including gastric and endometrial carcinoma [22,23]. Several studies have revealed substantial rates of receptor discordance between primary tumors and residual, recurrent, or metastatic disease, including shifts between HER2-0, HER2-low, and HER2-positive categories after neoadjuvant chemotherapy [19]. HER2 instability has direct clinical implications, as changes in HER2 status can alter therapeutic eligibility, allowing some patients previously considered ineligible to become candidates for ADCs [20,21].

In this narrative review, we synthesize current evidence on HER2-low and HER2-ultralow breast cancer, with a focus on HER2 status across disease evolution and under the selective pressure of systemic treatment. We summarize definitions, assessment challenges, and the reported frequency and patterns of HER2 category shifts. Furthermore, we discuss the practical implications for retesting and treatment selection in the era of ADCs.

## 2. Materials and Methods

A targeted literature search was conducted across PubMed, Scopus, and Web of Science, focusing on publications from 2020 to 2025. A small number of older key studies were also included for historical context and mechanistic insight. The search prioritized studies addressing HER2-low and HER2-ultralow breast cancer from pathological, biological, and clinical perspectives, including data on HER2 assessment, temporal shifts in HER2 category, and implications for therapy selection. Eligible studies included original research articles, phase II–III clinical trial reports, large retrospective cohorts, and review papers published in peer-reviewed journals. Case reports, small case series, conference abstracts without full datasets, and studies lacking a clearly described methodology for HER2 evaluation by IHC or in situ hybridization (ISH) were excluded. All included articles were reviewed in full for data extraction and narrative synthesis.

## 3. Results

### 3.1. Definition and Assessment

**Figure 1:**
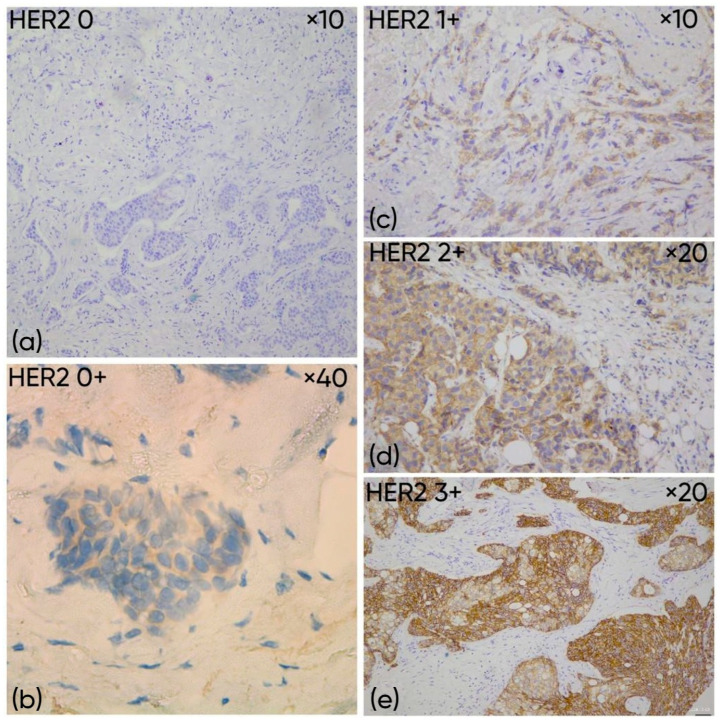
Representative examples of HER2 immunohistochemistry (IHC) scores in breast carcinomas. (**a**) HER2-0: no membranous staining in invasive tumor cells (objective ×10), (**b**) HER2-ultralow (IHC 0+): faint, incomplete membranous staining in ≤10% of invasive tumor cells (objective ×40); (**c**) HER2-low (IHC 1+): faint, incomplete membranous staining in >10% of invasive tumor cells (objective ×10), (**d**) HER2 2+: weak to moderate complete membranous staining in >10% of invasive tumor cells (objective ×20); (**e**) HER2 3: strong, complete membranous staining in >10% of invasive tumor cells (objective ×20).

HER2-low breast cancers are defined as tumors that do not meet the criteria for HER2 positivity but show detectable low-level membranous HER2 expression on IHC. In line with current American Society of Clinical Oncology/College of American Pathologists (ASCO/CAP) reporting criteria, this category includes tumors scored IHC 1+ or IHC 2+ with negative ISH findings [2,3,4,5,6,7,8,9,10]. To frame the HER2-low concept within routine scoring, HER2 IHC is assessed across a continuum. IHC 0 indicates no membranous staining, while HER2 0+ shows incomplete staining in ≤10% of tumor cells. IHC 1+ corresponds to faint and incomplete membranous staining in >10% of tumor cells. IHC 2+ is an equivocal category, typically reflecting weak-to-moderate complete membranous staining in >10% of tumor cells and requires ISH for final classification. IHC 3+ is defined by intense, complete, circumferential membranous staining in >10% of tumor cells and is considered HER2-positive [1,4,5] (Figure 1).

Importantly, a tumor can still be classified as triple-negative despite showing HER2-low characteristics, because this pattern is considered HER2-negative under ASCO/CAP criteria. Thus, the triple-negative breast cancer label is driven by estrogen and progesterone receptor negativity together with the absence of HER2 positivity, rather than by a complete lack of any HER2 signal [24,25].

### 3.2. HER2-Low as a Biological and Diagnostic Continuum

HER2-low breast cancer initially emerged as a diagnostic category defined by IHC criteria; however, accumulating molecular and functional data increasingly support the view that HER2-low represents a biologically meaningful intermediate state along a continuous spectrum of erb-B2 receptor tyrosine kinase (ERBB2) expression and signaling. Rather than constituting a discrete entity, HER2-low tumors appear to occupy a dynamic zone between complete absence of HER2 expression and HER2-overexpression-driven oncogenic addiction, with important implications for tumor biology, therapeutic vulnerability, and phenotypic instability [26].

From the perspective of intrinsic molecular subtypes, HER2-low tumors are unevenly distributed across breast cancer classes. They are most frequently observed within hormone receptor-positive disease, particularly luminal A and luminal B subtypes, whereas they are less common in basal-like tumors [26]. Nonetheless, a non-negligible fraction of triple-negative breast cancers also fall within the HER2-low category, highlighting the heterogeneity of this group. Compared with HER2-negative tumors, HER2-low cancers show a greater association with luminal differentiation, estrogen receptor signaling, and lower proliferative indices, suggesting that low-level HER2 expression may be linked to broader transcriptional programs characteristic of luminal biology rather than representing a random technical finding [2,27,28].

At the genomic level, HER2-low tumors are defined by the absence of ERBB2 amplification; however, they frequently exhibit detectable ERBB2 mRNA expression, placing them quantitatively between HER2-0 and HER2-amplified tumors. Large genomic studies have demonstrated that HER2-low tumors do not harbor a unique or pathognomonic mutational landscape; instead, their genomic profiles largely overlap with those of HER2-negative breast cancers, with recurrent alterations in genes such as *PIK3CA*, *TP53*, *MAP3K1*, and *GATA3*, depending on hormone receptor status. However, subtle differences in ERBB2 transcript abundance and the co-expression of ERBB family members suggest that HER2-low tumors may sustain a basal level of HER2-related signaling competence that is absent in truly HER2-null tumors [2,26].

Transcriptomic and proteomic data further reinforce the view of HER2-low as a biological intermediate. Relative to HER2-0 disease, HER2-low tumors show a modest enrichment of HER2-associated gene signatures and downstream signaling activity, particularly within the PI3K/AKT and MAPK pathways. Importantly, although this signaling is typically insufficient to confer classic HER2 oncogene addiction, it may provide survival advantages or modulate responsiveness to systemic therapies. Ligand-dependent heterodimerization between HER2 and other ERBB receptors, such as EGFR or HER3, likely plays a central role in sustaining this low-intensity signaling state, reinforcing the notion that HER2-low biology is context-dependent rather than driven by gene amplification alone [28].

From a biological standpoint, HER2-ultralow tumors can be viewed as a natural extension of the continuous spectrum of HER2 expression in HER2-negative disease, suggesting a state in which HER2-related signaling may persist at very low expression levels. In this setting, ultralow does not imply an ERBB-driven phenotype; rather, it likely reflects minimal receptor availability shaped by intratumoral heterogeneity, fine regulation of expression at the single-cell level, and context-dependent crosstalk within the ERBB network. As such, its biological relevance lies more in gradual variation and continuity than in delineating a distinct molecular entity [29].

Taken together, these observations support the view that HER2-low and, by extension, HER2-ultralow tumors are best understood as a biologically and diagnostically dynamic state emerging from the intersection of intrinsic disease biology, signaling context, and technical assessment variability. Furthermore, clinical experience with trastuzumab deruxtecan has demonstrated that ERBB2 amplification is not an absolute prerequisite for therapeutic benefit. In selected settings, even low-level HER2 expression can suffice for HER2 to serve as an anchoring target for an ADC, enabling intracellular cytotoxic payload delivery and broadening the practical implications of the HER2 expression continuum [2,26,30].

### 3.3. The Boundary Between HER2-Negative and HER2-Low

From a pathology perspective, this concept of HER2-low has emerged gradually, as successive guideline updates and accumulating clinical data have shifted the focus from a strict positive/negative result to recognition of an intermediate, low-expression range. This evolution has brought HER2-low to the forefront as both a biologically and clinically relevant label and a diagnostic challenge, particularly in ensuring reproducible distinction between HER2-0 and HER2-low in routine practice [4,5].

In daily practice, one of the most challenging aspects of HER2 assessment is distinguishing between IHC scores of 0 and 1+. Traditional HER2 testing algorithms were primarily designed to separate clearly HER2-positive tumors (IHC 2+ with positive ISH findings and IHC 3+) from HER2-negative cases; thus, pathologists and laboratories were less focused on refining the lower end of the scoring spectrum. As a result, very weak, incomplete, or focal membranous staining can be interpreted differently between observers, and even small variations in pre-analytical factors (such as fixation time or cold ischemia), the antibody clone used, or detection systems may shift a case across the 0/1+ threshold [4,5,19,20].

An additional area of uncertainty at the lower end of the HER2 spectrum is the emerging concept of HER2-ultralow. In practical terms, this label is generally used for tumors that remain classified as HER2-negative, yet show minimal, faint, and typically incomplete membranous staining in a very small proportion of invasive tumor cells, findings that fall below the conventional threshold for IHC 1+ [4,5,14,19,20,21]. The term HER2-ultralow was introduced to refine reporting within the HER2-negative continuum and better capture cases clustered around the IHC 0/1+ decision boundary. The clinical interest in this subgroup has increased as contemporary trials have begun to define and include it explicitly [21,22]. In the DESTINY study, “ultralow” eligibility has been operationalized as IHC 0 with detectable membrane staining, extending the evaluation beyond the traditional HER2-low definition [14,31]. However, terminology and implementation remain heterogeneous across institutions, and the approach has not been fully harmonized in routine guidelines in the same way as the established IHC scoring system. For now, HER2-ultralow should be viewed as a descriptive term for the lowest detectable HER2 expression level, with its reporting still evolving alongside ongoing clinical studies [32,33].

### 3.4. HER2-Low After Chemotherapy

In routine practice, the HER2 score should not be treated as a fixed label. Paired-sample studies have shown that HER2 expression can change over time under systemic treatment pressure and during disease progression, most often around the HER2-negative boundary between HER2-0 and HER2-low [Table 1] [19]. Miglietta et al. (2021) provided early, quantitative evidence of this instability in matched primary and relapse specimens, reporting an overall HER2 discordance of 38%, largely driven by bidirectional switching, alongside smaller fractions of changes involving HER2-positive disease [18]. In parallel, the DESTINY program made the practical consequences of this borderline range very clear at scale. Although these trials were designed to test treatment efficacy, central reassessment during screening demonstrated that classification at the 0/low interface is frequently not reproducible across testing contexts [13,31]. In the DESTINY-Breast04 trial, local and central assessments agreed on HER2-low status in approximately 78% of cases, and in DESTINY-Breast06, 64% of tumors previously reported as HER2 IHC 0 locally were classified as HER2-low/ultralow on central testing [13,31].

One of the earliest large-scale demonstrations that HER2 is not stable under systemic treatment came from Niikura et al., who analyzed a nationwide registry and showed that a substantial subset of tumors initially classified as HER2-positive were reclassified as HER2-negative after neoadjuvant chemotherapy. Although the HER2-low category did not exist at that time, these findings provided early evidence of clinically relevant HER2 conversion and laid the groundwork for later studies focusing on low-level HER2 expression [34]. Within the HER2-negative spectrum, subsequent neoadjuvant paired-sample cohorts have reinforced that most variability concentrates around the HER2-0/HER2-low boundary. Zhu et al. found frequent category changes between primary and residual disease, including substantial proportions of HER2-0 cases shifting to HER2-low and HER2-low cases reverting to HER2-0 among non-pCR cases, supporting the concept that low-level HER2 expression is dynamically reclassified after NAT [35]. Consistently, Kang et al. reported a high discordance rate after neoadjuvant chemotherapy, with prominent bidirectional transitions between HER2-0 and HER2-low, while also documenting smaller fractions of shifts involving HER2-positive conversion [36].

Two additional neoadjuvant series by Shang et al. and Sung et al. reported overall inconsistency rates in a similar range and, importantly for interpretation, suggested that loss of low-level expression (HER2-low to HER2-0) can be at least as common as gain (HER2-0 to HER2-low) in certain datasets, although the dominant direction varies across cohorts and subgroups [37,38]. Schettini et al., focusing on an HR-positive/HER2-negative paired-sample cohort, likewise observed frequent reclassification around the 0/low threshold, aligning with the broader message that HER2-negative continuum is not biologically or diagnostically static [27].

Finally, beyond neoadjuvant chemotherapy, real-world datasets have documented receptor–phenotype discordance between primary and metastatic disease at scale, as in the French ESME cohort, supporting the notion that HER2 reclassification can also reflect tumor evolution and sampling context progression, rather than solely treatment-related effects [39].

Biologically, therapy-associated shifts in HER2 status are most plausibly driven by treatment-imposed selective pressure, which can enrich tumor cell populations with lower ERBB2 amplification or induce adaptive reduction in membranous HER2 (including transcriptional downregulation or receptor internalization). However, the relative weight of these mechanisms across clinical cohorts remains incompletely defined and an active area for translational investigation [40,41].

### 3.5. Intratumoral Heterogeneity

**Figure 2:**
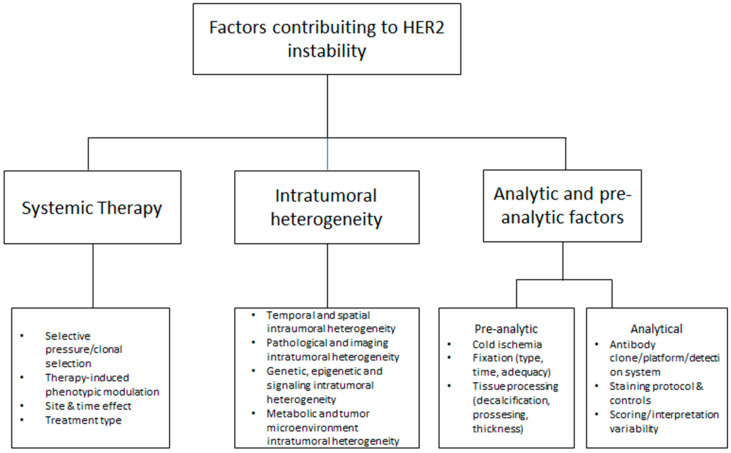
Conceptual framework summarizing key drivers of HER2 status instability: systemic therapy effects, intratumoral heterogeneity, and pre-analytical/analytical testing factors.

Another mechanism, beyond the selective pressure of systemic therapy, that may contribute to apparent HER2 instability is intratumoral heterogeneity (ITH) (Figure 2). ITH denotes the coexistence, within a single breast carcinoma, of distinct cellular subpopulations and micro-niches that differ in molecular profile, phenotype, and behavior, with direct implications for biomarker assessment, prognosis, and treatment response [42]. From an integrated perspective, ITH encompasses temporal heterogeneity (the evolution of tumor features over time due to natural progression and therapy-driven selection), spatial heterogeneity (regional differences within the tumor and between primary and metastatic sites), genetic heterogeneity (subclonal variation in somatic mutations and copy-number alterations), epigenetic heterogeneity (divergent DNA methylation and chromatin states shaping transcriptional programs without changes in DNA sequence), signaling heterogeneity (uneven activation of oncogenic and stress-response pathways), and metabolic heterogeneity (distinct metabolic adaptations driven by oxygen and nutrient gradients). Importantly, ITH also includes tumor microenvironment (TME) heterogeneity, referring to spatially variable immune and stromal composition, vascularization, and extracellular matrix features that can modulate clonal fitness and influence protein expression patterns [43,44]. These ITH dimensions provide a direct mechanistic link to HER2-low instability. Spatial heterogeneity can yield different HER2 scores when biopsy and resection samples do not overlap, or when primary and metastatic lesions capture distinct tumor areas. In parallel, genetic heterogeneity and TME-driven heterogeneity can shift the fraction of low-expressing cells and local expression states over time and across sites, leading to discordant HER2 categorizations between primary and metastasis on re-biopsy. Accordingly, this variability is particularly relevant near the HER2-0/HER2-low decision boundary, where limited sampling may capture different tumor regions and generate discordant results between core biopsy and the surgical specimen [42,43,44,45,46,47].

### 3.6. Pre-Analytical and Analytical Variability in Breast Cancer

Beyond biological mechanisms, HER2 status instability between core biopsies and surgical specimens may also reflect a combination of pre-analytical and analytical factors, which become particularly critical when low HER2 expression lies at the 0 vs. 1+ decision boundary [4,5]. Pre-analytically, major sources of variability include cold ischemia time (delay of fixation), the type of fixative and duration of fixation (typically 10% neutral buffered formalin, within guideline-recommended windows), tissue size and thickness (especially in large specimens with uneven fixative penetration), procedures that alter antigenicity (decalcification, suboptimal processing), block/slide storage conditions, and repeated recuts [48,49]. Analytically, variability may arise from the use of different platforms and assay reagents (primary antibody, detection chemistry, antigen retrieval), differences in instrument calibration and maintenance, lot-to-lot variation, incomplete assay validation or revalidation, and inconsistent implementation of internal/external controls and quality assurance programs. In this context, the choice of HER2 IHC assay and antibody clone is not trivial: differences in antibody affinity or epitope recognition, as well as in manufacturer-defined antigen retrieval conditions, can shift signal intensity in cases with weak staining. This shift may affect categorization around the boundary between IHC 0 and 1+ and, in borderline cases, give the impression of conversion between core biopsy and resection (Table 2) [50]. An additional layer is the interpretative component (interobserver reproducibility of IHC scoring, area selection, borderline cut-offs, and ISH counting/interpretation criteria), which can shift a case with very faint staining from one category to another. Accordingly, ASCO/CAP guidance and CAP reporting tools emphasize rigorous standardization of pre-analytical and analytical steps and, where feasible, documentation of key parameters (e.g., cold ischemia time and fixation) to minimize variability that can mimic true changes in HER2 status [4,5,49,51].

### 3.7. AI and HER2-Low Instability

Emerging data indicate that AI-assisted assessment of HER2 IHC can improve scoring reproducibility, with the clearest benefit at the most problematic threshold for HER2-low classification, distinguishing IHC 0 from 1+. Wu et al. reported that AI assistance improved the accuracy and consistency of interpreting HER2 IHC scores 0 and 1+ and reduced observer-related variability, supporting its role as a practical decision-support tool in borderline cases [55].

However, implementation requires careful attention to limitations, especially those relevant to HER2-low/ultralow. Real-world performance can be affected by domain shifts, including differences in staining intensity, fixations, antibody clones, scanning devices, and image compression, as well as the fact that low-level membranous signals are inherently subtle and spatially heterogeneous. Quality-assurance studies in routine settings have also highlighted pitfalls in discordant cases and remind us that digital tools may not uniformly reduce variability unless carefully calibrated and monitored within the local workflow [47,56,57,58].

Accordingly, the CAP emphasizes that computational pathology tools, including quantitative image analysis and AI, should be implemented within a rigorous validation and quality assurance framework and serve as decision support rather than replace the pathologist’s interpretation. In this context, decision support refers to validating the digital pathology system used for primary diagnosis, validating the HER2 quantitative/AI workflow in the intended clinical context, and establishing ongoing quality control, performance monitoring, and clear reporting practices [59].

## 4. Conclusions

The recognition of HER2-low and ultralow status supports a more nuanced, clinically actionable view of HER2 expression. Because HER2 status may vary over time and between the primary tumor and metastatic lesions, driven by treatment-related selective pressure and intratumoral heterogeneity, and by pre-analytical and analytical variability, HER2 retesting, when clinically appropriate, can identify patients previously deemed ineligible who may become candidates for targeted therapies. Rigorous standardization and transparent reporting remain essential, particularly at the challenging IHC 0 vs 1+ boundary.

## 5. Future Directions

Accurately identifying very low levels of HER2 expression has become clinically important in the era of HER2-directed ADCs. As HER2-low/ultralow status may vary over time, biomarker reassessment is increasingly relevant across the disease course. Observed changes can reflect spatial and temporal intratumoral heterogeneity, therapy-related effects, and pre-analytical and analytical variability. Thus, future strategies should move toward a more systematic approach to HER2 retesting whenever new clinically meaningful tissue becomes available, including local recurrence, metastatic progression, and residual disease after neoadjuvant therapy. Such an approach may reduce misclassification at the IHC 0/1+ boundary and help identify patients who may become eligible for HER2-targeted ADCs despite prior HER2-negative results.

To make retesting actionable and comparable across centers, research should prioritize standardized testing pathways (tissue handling and fixation, assay selection, on-slide controls, and reporting) and prospectively evaluate paired sampling across timepoints and sites. In parallel, the role of AI-assisted scoring and complementary quantitative methods should be explored primarily as tools to improve reproducibility at low expression thresholds, but only within robust validation and quality assurance frameworks. Ultimately, aligning retesting practices with rigorous analytical standards and transparent reporting is essential for translating the HER2-low/ultralow concept into consistent patient selection and equitable access to effective therapies.

## Figures and Tables

**Table 1:** Key studies on HER2 status instability after neoadjuvant therapy in breast cancer.

Author, Journal, Year of Publication	No. of Patients	Global Discordance %	HER2-0 → HER2-Low	HER2-Low → HER2 0	HER2-Positive → HER2-Negative	Conclusions
Miglietta et al., njp Breast Cancer, 2021 [18]	*n* = 291 (patients with residual disease)	38%	8.9%	14.8%	HER2-negative → HER2 positive 2.4% HER2-positive → HER2-negative 0.3%	HER2-low patients were associated with a lower rate of pathologic complete response.
Niikura et al., Annals of Oncology, 2016 [34]	*n* = 12 758	7.4%	N/A	N/A	HER2-positive → HER2-negative 21.4; HER2-negative → HER2-positive 3.4%	HER2 status can change after neoadjuvant chemotherapy, predominantly loss of HER2 positivity
Zhu et al., BJC, 2023 [35]	*n* = 1797 (patients with residual disease)	19.53%	39.5%	14.3%	HER2-positive → HER2 negative 7.3%	In this study, HER2 mainly gain expression.
Kang et al., EJC, 2023 [36]	*n* = 1288	36.5%	29%	32%	HER2-negative → HER2 positive 12.4%	After neoadjuvant chemotherapy, HER2 status is frequently reclassified, in this case, with similar bidirectional shifts.
Shang et al., Frontiers in Oncology, 2023 [37]	*n* = 775	21.42%	6.45%	10.19%	HER2-positive → HER2-negative 1.68% HER2-negative → HER2-positive 2.3%	HER2 status is frequently reclassified after neoadjuvant therapy, mainly with loss of low-level expression.
Sung et al., Cancer Res Treat, 2025 [38]	*n* = 670	21.2%	11.5%	2.7%	HER2-positive → HER2-negative 3.1% HER2-negative → HER2-positive 3.9%	HER2-low status is associated with a lower pathologic complete response and a higher residual cancer burden class after neoadjuvant therapy
Schettini et al., ESMO Open, 2024 [27]	*n* = 186	30%	25%	34%	N/A	HER2 in likely to lose expression than to gain it.

**Table 2:** Commonly used HER2 IHC antibodies/assays; typical platforms and antigen retrieval.

Antibody Clone/Assay	Typical Platform (s)	Antigen Retrieval (Relative, Manufacturer)
4B5 (PATHWAY/VENTANA HER2) [52]	Ventana/Roche BenchMark	HIER, alkaline/high-pH (Ventana CC1)
HercepTest^TM^ (Dako/Agilent) [53]	Dako/Agilent Autostainer Link + PT Link	HIER, Low pH epitope retrieval solution (kit-based, heated retrieval)
A0485 (polyclonal rabbit anti-HER2; commonly LDT) [50]	Various (commonly Dako/Agilent workflows)	HIER required; typically low-pH or high-pH after local optimization
SP3 (rabbit monoclonal antibody; commonly LDT) [50]	Various (platform-dependent)	HIER, often alkaline/high-pH
CB11 (Bond Oracle^TM^ HER2 IHC System; Leica) [54]	Leica BOND automated system	HIER, citrate/low-pH (Leica ER1; protocol default).

HIER, heat-induced epitope retrieval. LDT, laboratory-developed test (locally optimized protocol; conditions may vary by platform and require validation). CC1, Cell Conditioning 1. ER1, Epitope Retrieval Solution 1.

## Data Availability

No new data were created or analyzed in this study.

## Abbreviations

The following abbreviations are used in this manuscript:

IHC	Immunohistochemestry
ISH	In situ hybridization
HR	Hormone receptor
ADC	Antibody-drug conjugate
T-DXd	Trastuzumab deruxtecan
ASCO/CAP	American Society of Clinical Oncology/College of American Pathology
NAT	Neoadjuvant therapy
ITH	Intratumoral heterogeneity
TME	Tumor microenvironment
AI	Artificial intelligence
